# Comparison of Lipid and Palmitoleic Acid Induction of *Tribonema minus* under Heterotrophic and Phototrophic Regimes by Using High-Density Fermented Seeds

**DOI:** 10.3390/ijms20184356

**Published:** 2019-09-05

**Authors:** Wenjun Zhou, Hui Wang, Li Zheng, Wentao Cheng, Lili Gao, Tianzhong Liu

**Affiliations:** 1 Key Laboratory of Biofuels, Key Laboratory of Shandong Energy Biological Genetic Resources, Qingdao Institute of Bioenergy and Bioprocess Technology, Chinese Academy of Sciences, Qingdao 266101, China; 2 University of Chinese Academy of Sciences, Beijing 100049, China; 3 Key laboratory for Marine bioactive substances and modern analytical Technology, First Institute of Oceanography, State Oceanic Administration, Qingdao 266061, China; 4 Laboratory for Marine Ecology and Environmental Science, Qingdao National Laboratory for Marine Science and Technology, Qingdao 266071, China

**Keywords:** *Tribonema minus*, lipid, palmitoleic acid, heterotrophic fermentation, lipid enhancement

## Abstract

Palmitoleic acid, one scarce omega-7 monounsaturated fatty acid, has important applications in the fields of medicine and health products. *Tribonema* has been considered as a promising candidate for the production of palmitoleic acid due to its high lipid and palmitoleic acid content and remarkable heterotrophic ability. The high-density heterotrophic cultivation of *Tribonema minus* was conducted in this work, and the highest biomass of 42.9 g L^−1^ and a relatively low lipid content of 28.7% were observed. To further enhance the lipid and palmitoleic acid accumulation, induction strategies under two regimes of phototrophy and heterotrophy with different conditions were investigated and compared. Results demonstrated encouraging promotions both by heterotrophic and phototrophic ways, and the final lipid contents reached 41.9% and 49.0%, respectively. In consideration of the time cost, however, the induction under heterotrophic conditions was much more advantageous, by which the highest lipid and palmitoleic acid productivities of 1.77 g L^−1^ d^−1^ and 924 mg L^−1^ d^−1^ were obtained respectively, with the lipid yield on glucose of 0.26 g g^−1^.

## 1. Introduction

Palmitoleic acid (16:1, ω-7), one of omega-7 fatty acids, has been paid increasing attention in recent years due to the immune metabolic effects relevant to chronic metabolic diseases such as obesity, insulin resistance, nonalcoholic fatty liver disease (NAFLD), and atherosclerosis [1]. Palmitoleic acid acts as a lipokine modulating several metabolic processes in other tissues [2], reduces liver inflammation with NAFLD [3,4] and slows the progression of atherosclerosis in obese mice [5] by reducing inflammatory gene expression, lowering the production of inflammatory cytokines [5,6] and increasing differentiation to an anti-inflammatory phenotype [6,7]. Palmitoleic acid has also been described as a powerful blocker (of activity and expression) of stearoyl-CoA desaturase 1 (SCD-1), reducing insulin resistance to control obesity [2,8,9]. Therefore, it was identified as a specific lipid hormone or a “lipokine” responsible for linking the adipose tissue to systemic metabolism. Moreover, palmitoleic acid is considered to be an ideal material to produce biodiesel [10,11] and 1-octene [12,13]. Currently, the major sources of palmitoleic acid are sea buckthorn (*Hippophae rhamnoides* L.) berry containing about 40% of palmitoleic acid in its total lipid, macadamia nut containing 30% of palmitoleic acid in its total lipid and fish oil containing 10% of palmitoleic acid. However, the low lipid content (e.g., 3.5% for the sea buckthorn berry) or the poor agronomic characteristics of those sources inevitably results in the limitation of producing palmitoleic acid commercially [14,15]. Large scale planted oil crops, such as sunflower and jatropha, have been found only containing less than 2% of palmitoleic acid in their oil [16,17]. It has been also identified that most oleaginous microalgae could accumulate 8–25% of palmitoleic acid in the extracted fatty acids [18,19,20,21,22,23].

*Tribonema* sp., a filamentous oleaginous microalga, has been reported to accumulate a high content of lipid (40–60% of dry biomass) and palmitoleic acid (50% around of its lipid) [24,25,26,27]. Moreover, it was found that *Tribonema* sp. could grow heterotrophically by utilizing organic carbon sources, such as glucose, sucrose, and acetic acid [28]. This heterotrophic ability of *Tribonema* sp. is helpful to overcome the conditional limitations in a traditional microalgae cultivation such as location, season and light, and provides a feasible way to produce the palmitoleic acid in large scale [29,30]. However, previous work showed that the lipid content of *Tribonema* sp. under a heterotrophic condition was much lower than that under a phototrophic condition, probably caused by the changes of fatty acids metabolic pathways [29]. The same phenomenon was also observed in *Chlorella protothecoides*, a promising microalgal strain for lipids production, when a heterotrophic cultivation was employed [20,31,32].

Some nutrient regulation methods are widely identified effective to enhance the lipid accumulation of heterotrophic microalgae, such as depriving nitrogen and phosphorus, increasing the C/N ratio and salt stress, and adjusting the dosage of some metal ions. Shen et al. [33] reported that the highest fatty acid methyl ester (FAME) content (56%) of heterotrophic microalga *Chlorella vulgaris* fed on acetate was obtained in a nitrogen deficient medium, and the fatty acids productivity under this condition was three times greater than that under nitrogen sufficient conditions. The lipid production of *Chlorella regularis* cultivated heterotrophically was also promoted significantly under a nitrogen starvation stress [34]. In addition, providing appropriate concentrations of phosphorus, iron, and NaCl were also identified as positive measures for reinforcing the lipid accumulation of heterotrophic microalgae [32,33,35]. However, our previous work showed that the single stress of nitrogen, phosphorus, or iron performed only a slight influence on the lipid accumulation of heterotrophic *Tribonema minus* [29], indicating that the biosynthesis of lipid in heterotrophic *T. minus* cells is very likely associated with various nutrients in the medium. Therefore, the combined regulation needs to be further investigated.

Compared with the so called “one pot” method, which means that the cell proliferation stage and lipid induction stage were continuously carried out in one reactor without any replacement of the medium, two-stage cultivation mode (cultivating microalgae heterotrophically at the first stage and then transferring the cells into a new induction medium for subsequent cultivation at the second stage) has been usually used to promote the lipid accumulation of microalgae. Han et al. [21] cultivated three *Chlorella* species (*C. pyrenoidosa*, *C. ellipsoidea*, and *C. vulgaris*) heterotrophically at first and then the heterotrophic biomass was diluted and cultured under phototrophic conditions for the lipid accumulation. Results showed that the total lipid content of those *Chlorella* species increased from about 9% to 30% after four days of phototrophic cultivation. This method had a better performance on *Chlorella protothecoides*, according to the work published by Li et al. [36]. When the heterotrophy–photoinduction cultivation regime (HPC) was employed, the lipid content of *C. protothecoides* increased from 15.08% to 50.5% by 48 h of photo-induction, with the rapid decrease of starch and protein after 12 h of photo-induction. *Chlorella sorokiniana* was also tested using the same method and it was proved effective to promote the lipid production [37]. However, all above researches were focused on a unicellular *Chlorella* sp., no attempts of the lipid promotion for heterotrophic filamentous microalgae such as *Tribonema* sp. were reported.

In this study, *T. minus* was heterotrophically cultivated at high-density by a fed-batch cultivation in the bubble column. Then, additionally, in order to promote the lipid accumulation, the heterotrophic broth was transferred into two modes of phototrophy and heterotrophy with different conditions for the lipid induction. Results will reveal the characteristics of the lipid and palmitoleic acid accumulation in *T. minus* and then lead to an effective strategy to strengthen the lipid and palmitoleic acid contents for potential large-scale production.

## 2. Results

### 2.1. Cultivation and Lipid Accumulation of T. minus by Heterotrophy

#### 2.1.1. Growth

**Figure 1:**
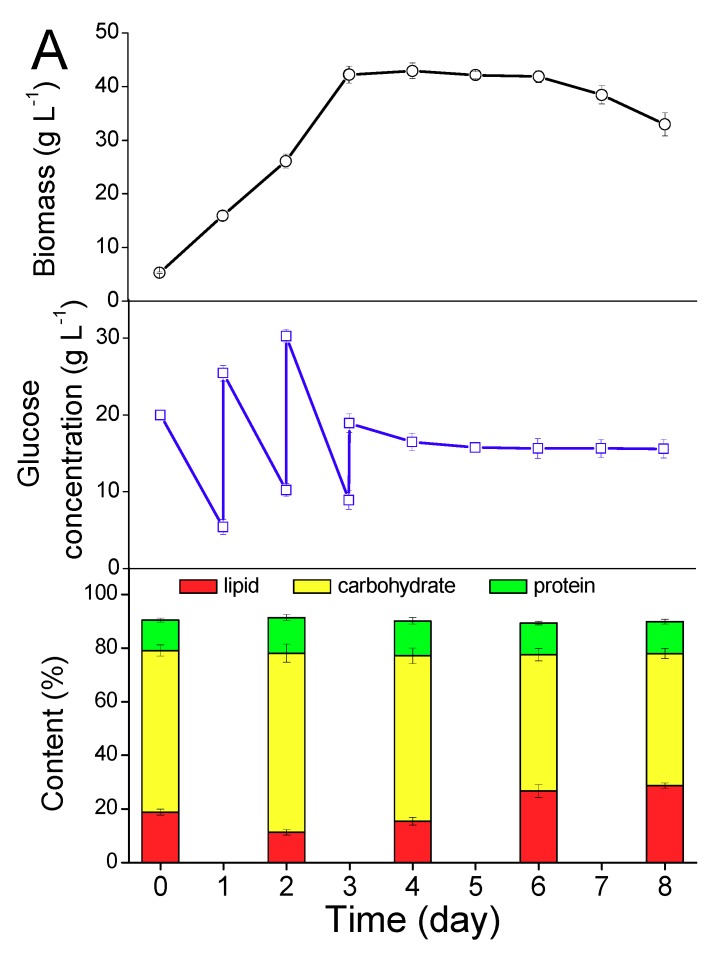

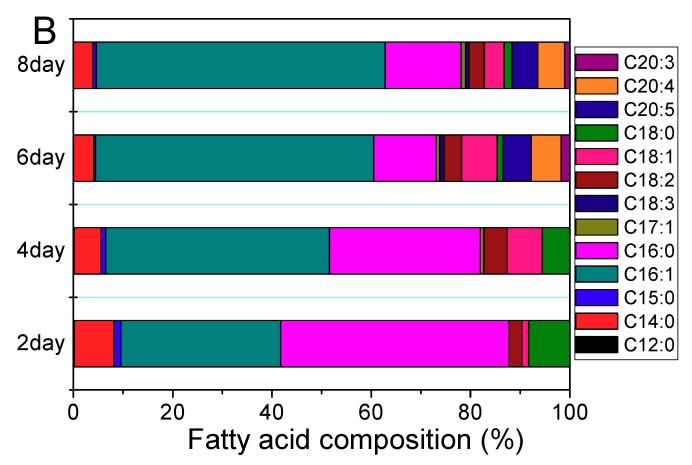
Growth, glucose consumption and cellular components (**A**), and fatty acid profiles (**B**) of heterotrophic *T. minus* in the bubble column. Values shown are averages of three replicates and error bars represent the standard deviations for the replicates.

A high-density cultivation of *T. minus* was carried out in the bubble column and the results were presented in Figure 1. The growth curve (Figure 1A) showed that *T. minus* grew very fast in the first three days, and the biomass density reached 42.2 g L^−1^ at day three. The highest biomass of 42.9 g L^−1^ was obtained at day four and the growth reached a stationary phase lasting for about three days, and then it entered the decline phase. The specific growth rate (μ) of the first three days was estimated as 0.693 d^−1^. Consequently, glucose was consumed quickly and fed at day one, two and three, and an average consumption rate of about 17 g L^−1^ glucose per day was estimated in the first three days, whereas during the stationary and decline phase, only a little of the glucose was consumed. The final glucose concentration was 15.6 g L^−1^ and the total consumption of glucose was calculated as 54.4 g L^−1^, which meant that the biomass yield on glucose was as high as 0.67 g g^−1^ (at day six, before the decline phase). This biomass yield on glucose was much higher than that of the heterotrophic *Chlorella* reported by Shi et al. (0.39–0.46 g g^−1^) [38].

#### 2.1.2. Lipid Accumulation

Lipid in the freeze-dried biomass was extracted with methanol-chloroform (2:1, *v*/*v*) and the lipid content was then calculated gravimetrically. The lipid contents at day two and day four were only 11.4% and 15.5%, respectively, but it reached a higher value of 26.7% and 28.7% at day six and day eight (Figure 1A). With the increase of the lipid, the carbohydrate content of the heterotrophic *T. minus* decreased from 66.7% at day two to 49.2% at day eight, whereas the protein content was relatively stable (around 12%). The lipid yield on the glucose of *T. minus* was estimated as 0.19 g g^−1^ (at day six) and the lipid productivity was calculated as 1.41 g L^−1^ d^−1^, 1.71 g L^−1^ d^−1^, and 1.05 g L^−1^ d^−1^, at day four, six, and eight, respectively, revealing that if this “one pot” cultivation method was used, the appropriate time to harvest *T. minus* is the sixth day, i.e., three days after the end of the exponential phase. Compared with the batch cultivation in our previous work [29], the highest biomass was improved from 30.8 g L^−1^ to 42.9 g L^−1^ by a fed-batch cultivation in this research, and the lipid productivity was also enhanced by 2.5 folds.

#### 2.1.3. Palmitoleic Acid Accumulation

Fatty acids in the lipid were converted into fatty acid methyl esters (FAMEs) and then analyzed by GC. The fatty acid profiles of *T. minus* cultivated at day two, four, six and eight were determined and displayed in Figure 1B. Agreeing with our previous work conducted by the phototrophic mode or heterotrophic mode in flasks [24,29], the dominated components in fatty acid profiles of *T. minus* fermented in the bubble column were the palmitoleic acid (C16:1) and palmitic acid (16:0), taking a total proportion of 68.7% to 78.1%.It is dramatic that the contents of the palmitoleic and palmitic acid changed significantly at different cultivation phases (*p* < 0.05). The amount of palmitoleic acid in total fatty acids of *T. minus* cells progressively increased from 32.2% (day two) to 58.2% (day eight), while that of the palmitic acid declined from 45.9% (day two) to 15.3% (day eight). Another interesting phenomenon was that the proportions of other saturated fatty acids such as C14:0 and C18:0 also continuously decreased throughout the fermentation process. This may be due to the activity of desaturases (e.g., Δ9 desaturase) that was enhanced with the extending of the cultivation. Consequently, the total unsaturated fatty acids (UFA) in total fatty acids at the end of the stationary phase constituted up to 81.8%, in contrast to 36.2% at the exponential phase. The palmitoleic acid productivity was estimated as 635 mg L^−1^ d^−1^, 958 mg L^−1^ d^−1^, and 611 mg L^−1^ d^−1^, at day four, six, and eight, respectively, meaning that for this “one pot” heterotrophic cultivation, the most feasible fermentation time for the palmitoleic acid production is six days, consistent with the result of the lipid production hereinbefore.

### 2.2. Lipid Induction under Different Regimes

The above work showed that the lipid content of *T. minus* by a heterotrophic cultivation was less than 30%, and there was still a great gap in comparison with the phototrophic cultivation (50–60%) [24]. For the potential industrial production of the palmitoleic acid, a fast lipid induction strategy is desired to greatly and efficiently increase the lipid and palmitoleic acid content. For this purpose, lipid inductions under two regimes of heterotrophy and phototrophy with different induction conditions were investigated. *T. minus* was heterotrophicly cultivated for four days at first as described in Section 2.1 and then harvested and washed as seeds for later experiments for lipid induction.

#### 2.2.1. Lipid Induction by Heterotrophy

**Figure 2:**
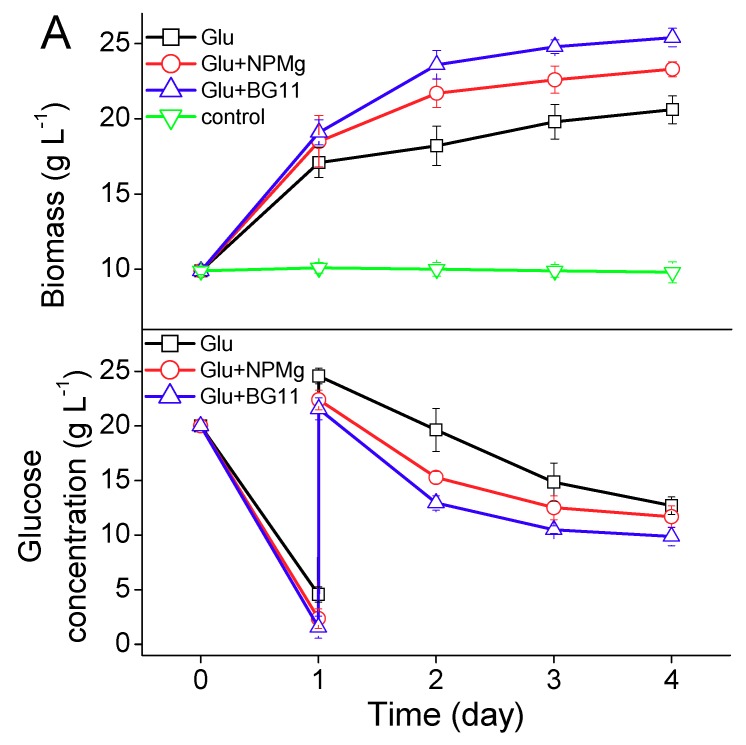

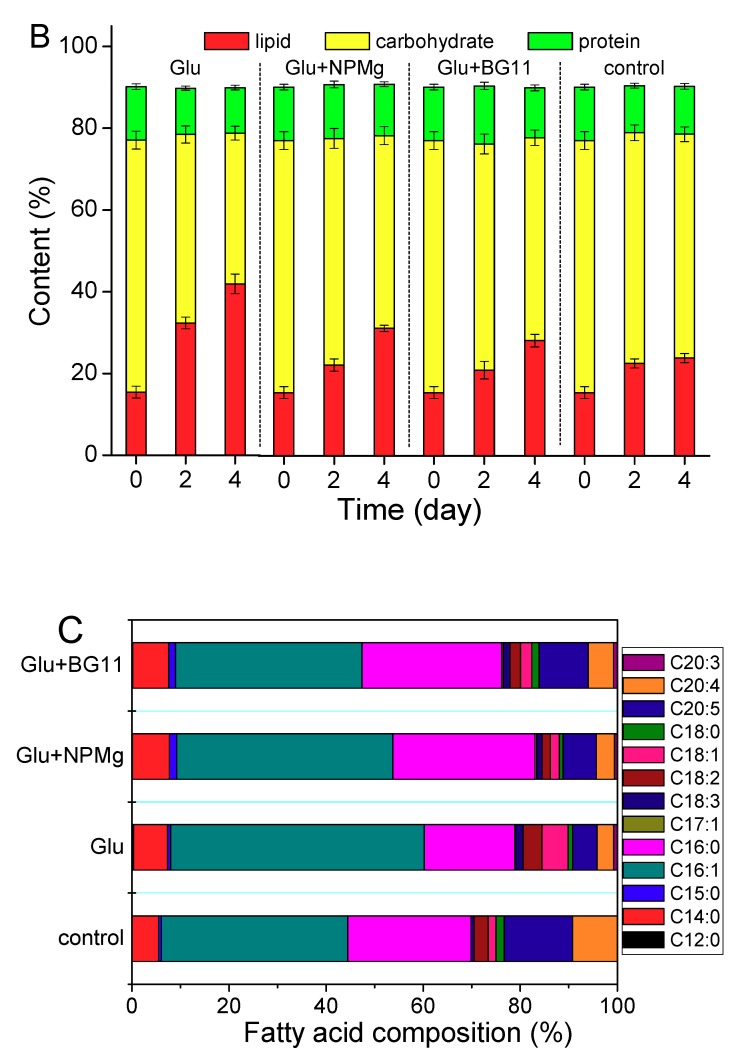
Growth curve and glucose consumption (**A**), cellular components (**B**) and fatty acid profiles (**C**) of *T. minus* in different mediums under heterotrophic regime. Values shown are averages of three replicates and error bars represent the standard deviations for the replicates.

The *T. minus* biomass was re-inoculated into three different heterotrophic mediums including the Glu medium (adding glucose in water), Glu+NPMg medium (adding glucose, N, P, and Mg in water), and Glu+BG11 medium (adding glucose in a BG11 medium) and one control medium (pure water). As presented in Figure 2A, *T. minus* grew well in those tested mediums except in the pure water, and the more abundant the nutrients provided, the faster the growth was and the higher the biomass achieved. The final biomass of *T. minus* after four days of induction cultivation was 20.6, 23.3, and 25.4 g L^−1^ in the Glu medium, Glu+NPMg medium, and Glu+BG11 medium, respectively, and the residual glucose in each medium was 12.7, 11.7, and 9.9 g L^−1^, respectively. According to the consumption of glucose, the final biomass yields on glucose were 0.40 g g^−1^, 0.48 g g^−1^, and 0.53 g g^−1^ (*p* < 0.05) for the medium Glu, Glu+NPMg, and Glu+BG11, respectively.

Conversely, the lipid content of *T. minus* in a different medium showed a great difference (Figure 2B). The lipid content of *T. minus* in a Glu medium fleetly increased from 15.5% at the beginning to 41.9% at day four, while other medium groups only performed a sluggish acceleration in the lipid accumulation, and the more comprehensive the nutrition was, the lower the lipid content increased. With the extension of the cultivation time, the carbohydrate content of *T. minus* decreased in all kinds of mediums, in which the Glu medium led to a greatest reduction from 61.6% at day 0 to 36.9% at day four. The final carbohydrate contents of *T. minus* in the Glu+NPMg medium and Glu+BG11 medium were 47.1% and 49.6%, respectively. A surprising result is that when *T. minus* was induced in pure water, although the biomass accumulation stopped, a sizable rise of the lipid content was observed. The lipid yield on the glucose of *T. minus* in the Glu medium was 0.26 g g^−1^, which was much higher than that in the Glu+NPMg medium (0.20 g g^−1^) and Glu+BG11 medium (0.19 g g^−1^) (*p* < 0.05). The lipid productivity was calculated as 1.77 g L^−1^ d^−1^, 1.44 g L^−1^ d^−1^, and 1.42 g L^−1^ d^−1^, in the Glu, Glu+NPMg, and Glu+BG11 medium, respectively, revealing that under the heterotrophic regime, the most appropriate induction medium to further reinforce the accumulation of lipid in *T. minus* cells was the solution only containing glucose.

**Figure 3:**
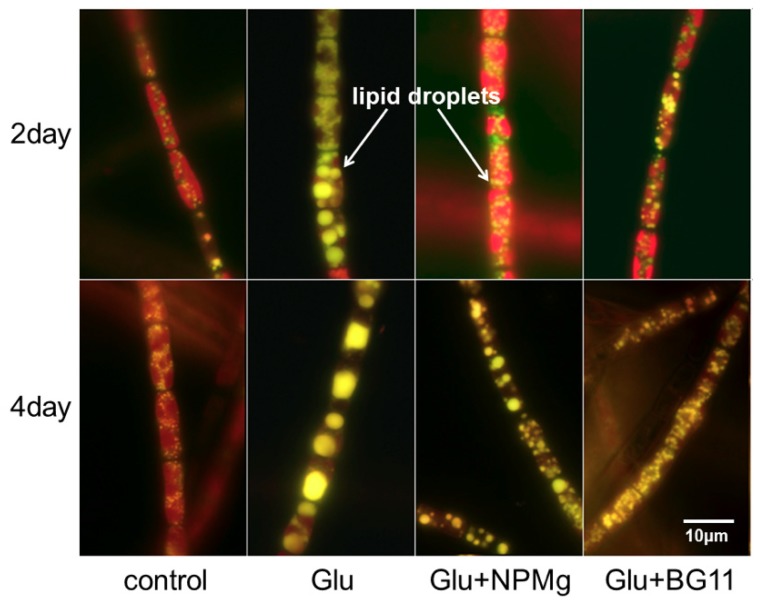
Fluorescence microscopy examination of *T. minus* in different mediums under the heterotrophic regime (stained with Nile Red).

According to the results presented in Figure 2C, the highest proportion of the palmitoleic acid in the fatty acid profile was also observed in the Glu medium, calculated as 52.2%, which resulted in a highest palmitoleic acid productivity of 924 mg L^−1^ d^−1^. The microscopic images of lipid droplets in *T. minus* cells stained by Nile Red were shown in Figure 3. It clearly demonstrated that lipid droplets of *T. minus* in the Glu medium were heaviest and greatest in all groups.

#### 2.2.2. Lipid induction by Phototrophy

**Figure 4:**
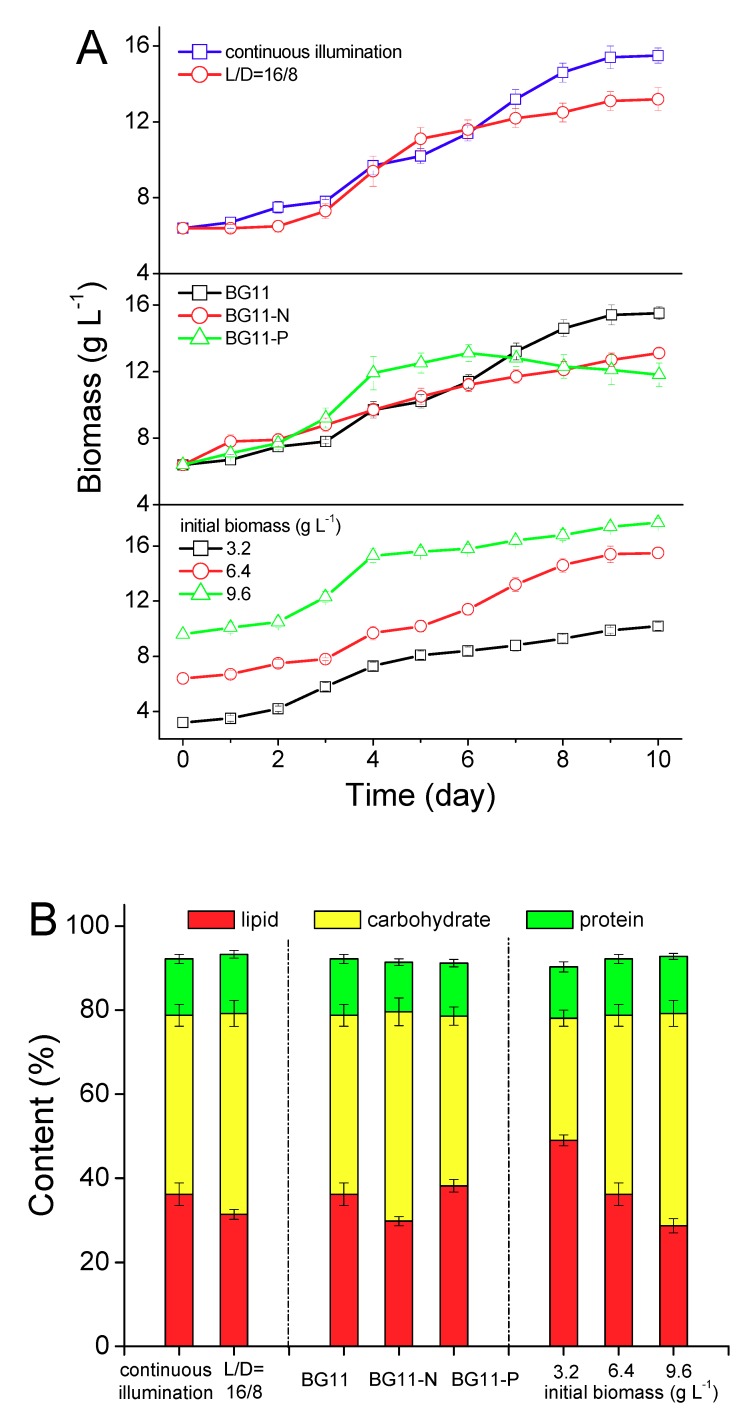

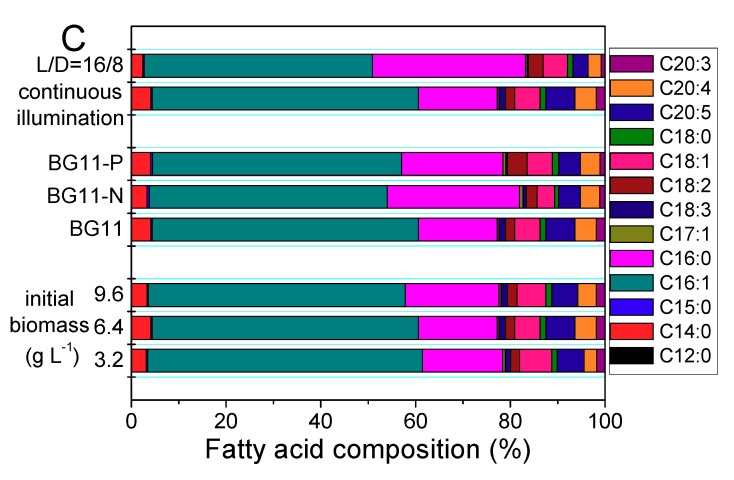
Growth curve (**A**), cellular components (**B**) and fatty acid profiles (**C**) of *T. minus* under the photoautotrophic regime. Values shown are averages of three replicates and error bars represent the standard deviations for the replicates.

In this process, heterotrophic *T. minus* seeds were re-inoculated into a phototrophic medium, and three factors were investigated including illumination, nutrients, and initial inoculation density. As presented in Figure 4A, there was a lag phase (about two days) for all the tested groups when heterotrophic *T. minus* seeds were re-inoculated into the phototrophic medium. Compared with the light/dark = 16h/8h (L/D = 16/8) group, continuous illumination was more appropriate for the recovering of the photosynthetic system in *T. minus* cells, and a higher biomass concentration (15.5 g L^−1^) was obtained. Both the N and P starvation resulted in a low final biomass density, even though the growth rate was motivated by the P starvation for a short time after the lag phase. In the initial inoculation biomass test, the highest final biomass of 17.7 g L^−1^ was reached when the inoculation density was 9.6 g L^−1^. However, in consideration of the biomass productivity, the optimal initial density was 6.4 g L^−1^, which led to a highest biomass productivity of 0.9 g L^−1^ d^−1^ under phototrophic conditions.

The lipid accumulation of *T. minus* re-cultivated phototrophically was significantly influenced by the induction conditions as plotted in Figure 4B (*p* < 0.05). The final lipid content under continuous illumination reached up to 36.2% from 15.5% at the beginning of the process, in comparison with 31.4% under a L/D = 16/8 illumination, indicating that reducing the illumination time weakened not only the biomass but also the lipid accumulation. In contrast with full strength of the BG11 medium, the absence of nitrogen in the medium was less advantageous for reinforcing the lipid accumulation of *T. minus*, which was consistent with the conclusion released by Guo et al. [25] under a phototrophic condition. Nitrogen starvation is generally identified as an effective regulation method for the lipid accumulation to various microalgae strains under phototrophy [39,40,41], but for *T. minus*, according to our present results, its inductive effect is relatively weak. This specific regulatory mechanism is still unclear, and further researches on the transcriptomics and metabolomics analysis under different N levels may lead to a clearer understanding. There was just a slight lipid promotion in the phosphorus deficient group, probably due to the longer stationary phase (in comparison with the BG11 group), in which lipid is the major product of biosynthesis in the *T. minus* cells. Among the three inoculation densities, the highest lipid content of 49.0% and lowest carbohydrate content of 29.1% were obtained simultaneously when the initial inoculation density was 3.2 g L^−1^, and the lipid content decreased when the initial inoculation density was enhanced. The lipid productivity was calculated as 0.45 g L^−1^ d^−1^, 0.45 g L^−1^ d^−1^, and 0.36 g L^−1^ d^−1^ with the three initial inoculation densities, respectively. This result agrees with our previous work [24], and the possible reason is that for the phototrophic microalgae, light is the sole energy to drive the fixation of CO_2_ and biosynthesis of biomolecules and more photons could be absorbed by algal cells under an appropriately diluted biomass density. By comparing the palmitoleic acid proportion in the fatty acid profiles under a heterotrophic induction (Figure 2C) and phototrophic induction (Figure 4C), a higher palmitoleic acid content was obtained under the latter one, while of which, reducing the illumination time, depriving of N or P in the medium, or inoculating excessive high initial biomass would result in the decrease of the palmitoleic acid proportion.

## 3. Discussion

*Tribonema* sp. has been reported to accumulate a high content of lipid (about 60% of dry biomass) and palmitoleic acid (about 50% of its lipid) when it was phototrophically cultivated [24,25,26,27], and this provides a new source for the production of the palmitoleic acid. However, the lipid content would significantly decrease to 20%, if the heterotrophic cultivation, which was considered to be much more feasible to scale up, was employed [29,30]. Several induction methods such as nitrogen deprivation [33,34] and photoinduction [21,36,37] are widely identified effective to enhance the lipid accumulation of the heterotrophic microalgae. In the present study, we focused on the lipid promotion of the heterotrophic *T. minus* under two regimes of phototrophy and heterotrophy with different inductive conditions. Results revealed that the lipid content was only 15.5% at day four and a little promotion (26.7% at day six and 28.7% at day eight) was observed at the end of the fed-batch fermentation without induction, indicating that for the heterotrophic cultivation of *T. minus*, a simple extension of fermentation is not a desirable means to improve the lipid accumulation, while the lipid content would be strongly enhanced if appropriate inductions were employed.

Under the heterotrophic induction conditions, the richness of nutrients in the medium played an important role in the lipid accumulation according to our results, and the more comprehensive the nutrition was, the lower the lipid content increased. The highest lipid content (41.9%) of *T. minus* was obtained in the medium only containing glucose. Results indicated that, under the comprehensive nutrients condition, the major product of biosynthesis in *T. minus* was the carbohydrate for the cell growth and reproduction, rather than the lipid which is generally synthesized as the energy storage chemical when microalgal cells are exposed to the adverse conditions. While a large amount of lipid would be synthesized if the *T. minus* cells were inoculated into the medium, which was deficient in various nutrients and only supplied with glucose as an essential carbon source. This was interesting because the previous work mainly focused on the single stress of nitrogen, phosphorus, iron, or salinity [32,33,34,35], and that only performed a slight influence on the lipid accumulation of heterotrophic *T. minus* [29]. A surprising result of this research is that a sizable rise of the lipid content was observed when *T. minus* was induced in pure water. A possible explanation is that the de-novo lipid biosynthesis would still be possible while the carbohydrate is consumed for respiration to maintain other biosynthetic pathways under the condition of extreme nutritional deficiency. Under the phototrophic induction condition, there was a lag phase (two days) for the growth of *T. minus*, and it is reasonable because the photosynthetic system, that is relatively feeble in heterotrophic seeds, needs to be rebuilt under phototrophic conditions and a certain time is necessary. The lipid content of *T. minus* also could reach 49% after 10 days of phototrophic induction, and this was similar to the results of the traditional phototrophic cultivation [25,26].

A brief comparison of the lipid productivity by the two lipid induction methods of phototrophy and heterotrophy was listed in Table 1. Both the two induction regimes were effective to enhance the lipid accumulation and resulted in a 3-fold increase of the total lipid content. The phototrophic induction had a better performance in the lipid and palmitoleic acid accumulation than the heterotrophic induction did, whereas a longer cultivation time was indispensably required for the phototrophic method, which correspondingly resulted in a little lower lipid and palmitoleic acid productivity. Moreover, the heterotrophic induction was independent of light and could be conducted at a high inoculation density, and these advantages made it more efficient for the lipid and palmitoleic acid production in less space occupation and more feasible in any climatic and seasonal location to scale up. In addition, it should be noted that the *T. minus* cells could be easily harvested by a gauze or sieve due to the filamentous morphology, and the renewal of the medium and in situ inoculation could be handled aseptically if an inner filter is installed in the fermenter. So, the operation process could be simplified, and the potential contamination problem could be avoided effectively. Therefore, rather than the phototrophic induction method, the heterotrophic induction was much more advantageous for the fermented *T. minus*, resulting in the highest lipid and palmitoleic acid productivities of 1.77 g L^−1^ d^−1^ and 924 mg L^−1^ d^−1^, respectively.

It seems that there is only a minor difference between the two lipid productivities of the “one pot” fed-batch fermentation (the highest lipid productivity was 1.71 g L^−1^ d^−1^) and the heterotrophic induction cultivation, and the former could be still useful for the production of the lipid and palmitoleic acid owing to its remarkable productivities. Actually, the major drawback of the “one pot” cultivation is the relatively low lipid content, and this will complicate the lipid extraction in a potential large-scale lipid production. However, the two-phase induction method will get over this limitation due to the highly enhanced lipid content.

## 4. Materials and Methods

### 4.1. Microalga and Culture Conditions

Filamentous microalga *T. minus* was purchased from SAG culture collection, University of Gottingen, and maintained in a BG11 medium [42]. Inoculum for the first stage of the heterotrophic cultivation was prepared in 250 mL flasks with 100 mL of a modified BG11 medium (Table 2) by adding glucose and urea according to our previous work on medium optimization [29], and then incubated in an orbital shaker with a stirring speed of 180 rpm and temperature of 27 °C for about three days. The pH value of the medium was maintained between 7 and 7.5 by adding 1 mol L^−1^ of NaOH or HCl solution in all cases throughout the study.

### 4.2. Experimental Design

Heterotrophic cultivation: Fed-batch cultivation was performed for eight days at 27 °C in a 1000 mL glass bubble column (covered with silver paper) with 800 mL of a modified BG11 medium, in which the glucose was fed once a day to maintain the glucose concentration at around 20 g L^−1^. The initial biomass was 5 g L^−1^, and the air flow rate was 0.5 vvm (air volume/culture volume/min).

Lipid induction: Prior to this process, the *T. minus* biomass was prepared by repeating the above fed-batch cultivation for four days to achieve the highest biomass (the first stage), and then the algal cells were harvested by a sterilized gauze and washed by sterile water three times carefully in a clean operating cabinet. The harvested *T. minus* biomass was subsequently used in the lipid induction process (the second stage) by using a 1000 mL glass bubble column with the 800 mL medium at 27 °C. The experimental arrangement for this process was listed in Table 3, and simply described as follows.

Lipid induction by heterotrophy: In order to investigate the combined regulation of various nutrients on the lipid accumulation, four different kinds of medium including the Glu medium, Glu+NPMg medium (with the same concentration of nitrogen, phosphorus, and magnesium to the BG11 medium), Glu+BG11 medium, and control medium were designed according to the abundance of the nutrients. The above harvested *T. minus* was re-suspended in the four different media and cultured in a glass bubble column covered with silver paper. The initial glucose concentration was 20 g L^−1^ and the glucose was batch-fed to maintain the concentration at around 20 g L^−1^ during the process.

Lipid induction by phototrophy: For this regime, three factors were investigated including the illumination, nutrient, and initial biomass. The illumination intensity was fixed at 100 μmol photons m^−2^ s^−1^, and the carbon source was CO_2_ enriched air by the ratio of 1%. The glass bubble column used in this section was not covered with silver paper.

### 4.3. Analytical Methods

The biomass was determined by a gravimetric method. 5 mL of broth (V) was taken from each cultivation per day. The samples were then filtered by pre-weighed 0.45 μm GF/C filter membranes (Whatman, DW_0_) and washed by distilled water three times. The membranes were dried to a constant weight at 105 °C and weighed (DW_1_). The dry weight (DW) was calculated as (DW_1_−DW_0_)/V [43]. The specific growth rate (μ) for the cultivation was calculated as (lnB_t_−lnB_0_)/(t−t_0_), wherein B_t_ and B_0_ were the biomass at time t and t_0_, respectively.

The lipid content was determined by the way according to Chen et al. [44]. The biomass from a 10 mL broth was filtered and freeze-dried. Approximately 50 mg of dried biomass was extracted with methanol-chloroform (2:1, *v*/*v*) after grinding, then the solution was separated into chloroform and aqueous methanol layers by adding the chloroform and 1% NaCl solution to make a final volume ratio of chloroform: methanol: 1% NaCl of 1:1:0.9. The chloroform layer was collected by a pipette and evaporated by nitrogen-blowing, and the lipid content was then calculated gravimetrically.

Fatty acids in the lipid were converted into fatty acid methyl esters (FAMEs) and then analyzed on a Varian 450GC (Varian Inc., Palo Alto, CA, USA). The lipid, containing a nonadecanoic acid (C19:0) added as an internal standard, was converted into FAMEs with the sulfuric acid/methanol (1:50, *w*/*v*) and incubated at 85 °C for 2.5 h. Nitrogen was used as a carrier gas during the FAMEs analysis on 450GC, and the injector temperature was set at 280 °C with an injection volume of 2 μL under a split mode of 10:1. The individual FAMEs were identified by a chromatographic comparison with authentic standards (Sigma, St. Louis, MO, USA) and calculated by the way reported by Chen et al. [44].

The Nile Red staining of microalgal cells was operated by the way according to Cooksey et al. [45], and the fluorescence examination was carried out on a fluorescence microscope (OLYMPUS BX35, Tokyo, Japan). The contents of the protein and carbohydrate were determined by the methods described by the national standards of the People’s Republic of China (GB 5009.5-2016 and GB/T 15672-2009, respectively).

The glucose concentration of the medium was analyzed by using a biosensor (SBA-40D, Jinan, China). The biomass yield on the glucose was calculated as (B_1_−B_0_)/(G_0_−G_1_), wherein B_0_ and G_0_ were the initial biomass and total glucose concentration, and B_1_ and G_1_ were the final biomass and residual glucose concentration, respectively. The lipid yield on glucose was calculated as (B_1_ × LC_1_−B_0_ × LC_0_)/(G_0_−G_1_), wherein B_1_ and B_0_ were the final biomass and initial biomass, respectively, LC_1_ and LC_0_ were the final lipid content and initial lipid content, respectively, and G_0_ and G_1_ were the total glucose concentration and residual glucose concentration, respectively.

The biomass productivity was calculated as (B_1_−B_0_)/T, wherein B_1_ and B_0_ were the final biomass and initial biomass, respectively, and T was the culture time. The lipid productivity was calculated as (B_1_ × LC_1_−B_0_ × LC_0_)/T, wherein B_1_ and B_0_ were the final biomass and initial biomass, respectively, LC_1_ and LC_0_ were the final lipid content and initial lipid content, respectively, and T was the culture time. The palmitoleic acid productivity was approximately calculated as LP × PC, wherein LP was the lipid productivity and PC was the palmitoleic acid content in the fatty acid profile.

### 4.4. Statistical Analysis

All the experiments were performed in parallel triplicates and repeated twice for validation, and data were denoted as a mean value ± SE (standard error). The statistical analysis of the data was performed using the SPSS (version 18.0, Chicago, IL, USA). A one-way analysis of variance (ANOVA) was used to evaluate the difference of the biomass and lipid content among the treatments at a confidence level of 0.05 and a LSD Post Hoc test for the ANOVA was applied.

## Figures and Tables

**Table 1:** Comparison between the phototrophic and heterotrophic methods to promote the lipid of *T. minus*. Data is presented as an average of three replicates with standard deviation.

Induction Methods	Phototrophy	Heterotrophy
Initial lipid content (% DW)	15.5 ± 1.4	15.5 ± 1.4
Final lipid content (% DW)	49.0 ± 1.3	41.9 ± 2.4
Maximum lipid productivity (g L^−1^ d^−1^)	0.45	1.77
Maximum palmitoleic acid content (% fatty acid profile)	58.0	52.2
Maximum palmitoleic acid productivity (mg L^−1^ d^−1^)	261	924

**Table 2:** Composition of a modified BG11 medium.

Modified BG11 Medium			
Glucose	20 g L^−1^	ZnSO_4_·7H_2_O	0.222 mg L^−1^
Urea	2 g L^−1^	CuSO_4_·5H_2_O	0.079 mg L^−1^
K_2_HPO_4_	400 mg L^−1^	MnCl_2_·4H_2_O	1.81 mg L^−1^
MgSO_4_·7H_2_O	375 mg L^−1^	Na_2_MoO_4_·2H_2_O	0.39 mg L^−1^
CaCl_2_·2H_2_O	180 mg L^−1^	Co(NO_3_)_4_·6H_2_O	0.0494 mg L^−1^
Na_2_CO_3_	100 mg L^−1^	H_3_BO_3_	2.86 mg L^−1^
Citric acid	30 mg L^−1^	Na_2_EDTA	5 mg L^−1^
Ammonium ferric citrate	24 mg L^−1^		

**Table 3:** Experimental arrangement for the lipid induction.

Regimes	Specific Conditions	
Heterotrophy (air flow rate of 0.5 vvm with pure air, initial biomass of 9.4 g L^−1^)	Glu medium (adding glucose in water)	
	Glu+NPMg medium (adding glucose, N, P and Mg in water)	
	Glu+BG11 medium (adding glucose in BG11 medium)	
	Control (pure water)	
Phototrophy (100 μmol photons m^–2^ s^−1^, air flow rate of 0.5 vvm with 1% CO_2_ in air)	Illumination test (BG11 medium and initial biomass of 6.4 g L^−1^)	Continuous
		Alternative (light/dark = 16 h/8 h)
	Nutrient test (continuous illumination and initial biomass of 6.4 g L^−1^)	BG11 medium
		BG11-N medium (N-free)
		BG11-P medium (P-free)
	Initial inoculation biomass test (continuous illumination and BG11 medium)	3.2 g L^−1^
		6.4 g L^−1^
		9.6 g L^−1^
